# Reactive Oxygen Species Stimulate Insulin Secretion in Rat Pancreatic Islets: Studies Using Mono-Oleoyl-Glycerol

**DOI:** 10.1371/journal.pone.0030200

**Published:** 2012-01-17

**Authors:** Marylana Saadeh, Thomas C. Ferrante, Ada Kane, Orian Shirihai, Barbara E. Corkey, Jude T. Deeney

**Affiliations:** Department of Medicine, Obesity Research Center, School of Medicine, Boston University, Boston, Massachusetts, United States of America; University of Ulster, United Kingdom

## Abstract

Chronic exposure (24–72 hrs) of pancreatic islets to elevated glucose and fatty acid leads to glucolipoxicity characterized by basal insulin hypersecretion and impaired glucose-stimulated insulin secretion (GSIS). Our aim was to determine the mechanism for basal hypersecretion of insulin. We used mono-oleoyl-glycerol (MOG) as a tool to rapidly increase lipids in isolated rat pancreatic ß-cells and in the clonal pancreatic ß-cell line INS-1 832/13. MOG (25–400 µM) stimulated basal insulin secretion from ß-cells in a concentration dependent manner without increasing intracellular Ca^2+^ or O_2_ consumption. Like GSIS, MOG increased NAD(P)H and reactive oxygen species (ROS). The mitochondrial reductant ß-hydroxybutyrate (ß-OHB) also increased the redox state and ROS production, while ROS scavengers abrogated secretion. Diazoxide (0.4 mM) did not prevent the stimulatory effect of MOG, confirming that the effect was independent of the K_ATP_-dependent pathway of secretion. MOG was metabolized to glycerol and long-chain acyl-CoA (LC-CoA), whereas, acute oleate did not similarly increase LC-CoA. Inhibition of diacylglycerol kinase (DGK) did not mimic the effect of MOG on insulin secretion, indicating that MOG did not act primarily by inhibiting DGK. Inhibition of acyl-CoA synthetase (ACS) reduced the stimulatory effect of MOG on basal insulin secretion by 30% indicating a role for LC-CoA. These data suggest that basal insulin secretion is stimulated by increased ROS production, due to an increase in the mitochondrial redox state independent of the established components of GSIS.

## Introduction

Obesity and diabetes have become increasing problems in the world, a direction that began about 30 years ago and continues in most countries. Diabetes develops in about 20% of obese individuals, and it is not known what differentiates those who develop diabetes from those who do not.

Obesity results from and sustains elevated circulating insulin. Insulin hypersecretion mostly thought to result from insulin resistance, has also been shown to cause insulin resistance as demonstrated in rodents overexpressing the human insulin gene [1] or treated with exogenous insulin [2], and by insulin infusion in humans [3]. This suggests the possibility that insulin hypersecretion may precede and drive the early stages of insulin resistance. The timing of these two metabolic impairments may be so inextricably linked that the order of events may never be elucidated, however, this demonstrates the importance of understanding both processes. Type 2 diabetes (T2D) occurs when hypersecretion fails to compensate for insulin resistance. Hypersecretion at basal glucose is an associated problem in that it reduces the effectiveness of stimulatory glucose. The mechanism for basal hypersecretion of insulin has received little attention.

High insulin secretion in the absence of stimulatory glucose can be caused *in vivo* by high fat feeding and mimicked *in vitro* by prolonged exposure to fatty acids (FA) [4], [5]. Although FA acutely enhance GSIS [6], chronic exposure of ß-cells to elevated levels of FA and glucose, designated as glucolipoxicity (GL), is a condition that mimics early type 2 diabetes in that it is characterized by increased basal insulin release and impaired GSIS [4].

In ß-cells, the toxic effects of lipids and glucose, alone or together have recently received abundant attention [7], [8], [9]. FA are non-toxic essential nutrients that circulate in the blood at levels of 0.1 to 1.0 mM complexed to albumin. The terms glucotoxicity, lipotoxicity and glucolipotoxicity have no generally accepted definition. In different studies, they refer to different combinations and concentrations of glucose and FA, FA chain length, FA saturation, and FA to albumin complexes [10], [11], [12], [13]. Clearly, excessive and non-physiological levels of glucose and FA, or saturated FA alone, induce ß-cell damage ultimately leading to apoptosis and cell death. Combinations that include physiological levels of mono-unsaturated FA, alone or together with saturated FA, and bound to albumin in appropriate ratio are not toxic to cells but do stimulate insulin secretion. This also occurs *in vivo* with a nutrient rich diet or in various models of obesity and diabetes. In this study we refer to extended exposure to physiological concentrations of FA and glucose as glucolipoxity as originally proposed by Prentki and Corkey [14].

Mono- and diglycerides are commonly added to commercial food products in small quantities. They act as emulsifiers, helping to mix ingredients such as oil and water that would not otherwise blend well, and as preservatives. They are often found in bakery products, beverages, ice cream, chewing gum, shortening, whipped toppings, margarine, and confections. One special mono-acyl-glycerol, 2-arachidonoyl-glycerol, is a full agonist of the cannabinoid receptors and thus classified as an endocannabinoid [15]. In this study, only MOG will be used, as it acutely stimulates insulin secretion and can be compared to oleate that does not stimulate basal insulin secretion. Mono-acyl-glycerides may be ingested or formed biochemically in the gut by release of FA from the 1 and 3 positions of triglyceride [16] and in cells via release of FA from diacylglycerol (DG), by diacylglycerol lipase or hormone sensitive lipase, and broken down by mono-acylglycerol lipase (MGL). Zawalich and co-workers documented that low concentrations (25–50 µM) of MOG enhance insulin secretion stimulated by a variety of agonists [17], [18] and 100 µM increased secretion at substimulatory glucose [17], an effect they attributed to inhibition of diacylglycerol kinase (DGK).

The insulin secretory process involves a combination of a Ca^2+^-dependent triggering pathway and an amplification pathway that requires a permissive level of Ca^2+^. Intracellular lipids have gained attention as an important part of the amplification pathway and as likely candidates to provide aberrant signals leading to impaired insulin secretion [4], [5], [19], [20]. The hypothesis being evaluated in this study is that if glucose and FA lead to accumulation of intracellular mediators resulting in time-dependent impaired insulin release, MOG may acutely elevate a similar set of mediators at basal glucose.

The advantage of the rapid MOG effect, supported by our data, is that GL-induced metabolic changes could be mimicked in a very short time without altered gene expression that occurs with the longer incubations (1–2 days) needed by elevated glucose and FA. Our data suggest three MOG-induced mediators of insulin secretion: cellular redox state reflected in the NAD(P)H∶NAD(P) ratio (redox), ROS and LC-CoA. Insight into these mediators could provide new targets to ameliorate early insulin hypersecretion and later impaired ß-cell function.

## Methods

### Ethics Statement

This study was carried out in strict accordance with the recommendations in the Guide for the Care and Use of Laboratory Animals of the National Institutes of Health. The protocol was approved by the Committee on the Ethics of Animal Experiments of the Boston University Medical Center (Boston University Medical Center Animal Welfare Assurance: A-3316-01).

### Islets

Islets were isolated from male Sprague-Dawley rats (150–250 g) [21]. Briefly, 15 ml of Hank's balanced salt solution containing 10 mM Hepes (pH 7.4), 3 mM glucose (HBSS) and 0.2 mg/ml collagenase P (2.3 units/mg) (Roche, Indianapolis, IN) was infused into the pancreas through the bile duct. The inflated pancreas was then excised and incubated for 20 min at 37°C. The digested pancreas was then shaken to release the islets, which were washed with HBSS supplemented with 0.1% BSA and further isolated by centrifugation through a Histopaque gradient (Sigma, St. Louis, MO) [21]. Human islets were obtained from the National Disease Research Interchange (www.ndriresource.org). Isolated islets were cultured overnight in RPMI 1640 containing 10% FBS, 50 IU/ml penicillin and 50 µg/ml streptomycin. Islets were dissociated in HBSS containing 20 mM Hepes, 3 mM EGTA, 3 mM glucose, 2.5% BSA and 0.001% trypsin at 37°C for 2.5 min [21], [22] and attached to cover slips with CELL-TAK or plated in multiwell culture plates mixed with matrigel (1∶1 ratio) as per product instructions (BD Biosciences, Bedford, MA).

### INS-1 832/13 cells

INS-1 832/13 cells [23] were cultured in RPMI media as above with the addition of 10 mM Hepes, 1 mM pyruvate and 50 µM ß-mercaptoethanol [4]. ß-Mercaptoethanol was routinely added to the RPMI media just before use.

### Lipid preparations

MOG was prepared as a DMSO stock, which was diluted into modified Krebs-Ringer bicarbonate buffer (KRB) at 37°C while vortexing. Oleate was dissolved in DMSO and complexed to either FBS (Invitrogen, Carlsbad, CA) or fatty acid free BSA (4∶1 molar ratio) at 56°C while vortexing. Final concentration of DMSO was 0.1%.

### Perifusion

Groups of 60 islets were placed in a column on top of a 1 cm bed of cytodex 3 beads and perifused at 37°C at a rate of 0.3 ml/min with KRB containing 3 mM glucose for 30 min [23]. After 30 min samples were collected at 15 sec intervals. Perifusate was changed as indicated in figure legends. KRB contained (in mM) 119 NaCl, 4.6 KCl, 5 NaHCO_3_, 2 CaCl_2_, 1 MgSO_4_, 0.15 Na_2_HPO_4_, 0.4 KH_2_PO_4_, 20 HEPES, 0.05% BSA, pH 7.4.

### Insulin secretion

Insulin secretion was measured in INS-1 cells grown for at least 3 days to approximately 0.25 million cells/well in 48-well plates. INS-1 cells were preincubated with RPMI containing 2 mM glucose without serum for 2 hrs prior to KRB incubation. INS-1 cells and dissociated islets in matrigel (4–5 thousand cells/well) were preincubated in low glucose KRB at 37°C for 30–45 min. They were then cooled on ice and cold KRB with test compounds was added. Cells were incubated for 30–60 min at 37°C, cooled and sampled for insulin release. Insulin was measured by radioimmunoassay (Millipore, Billerica, MA).

### Ca^2+^ determination

Intracellular Ca^2+^ was measured in cells using fura-2, AM (Invitrogen, Carlsbad, CA). Single cells from dissociated rat pancreatic islets were attached to glass bottom 35 mm dishes (MatTek, Ashland, MA) with CELL-TAK. The glass bottom dishes were mounted onto the stage of a Zeiss IM-35 fluorescence microscope and Ca^2+^ was monitored continuously, using a time-sharing fluorometer with output to a cooled CCD camera (IONOPTIX Corp., Boston, MA) [24].

### ROS determination

Cells were loaded for 45 min with 8 µM 5-(and-6)-chloromethyl-2′,7′-dichlorodihydrofluorescein diacetate, acetyl ester (CM-H2DCFDA) suspended in KRB containing 0.1% pleuronic acid, followed by two 15 min washes in KRB. DCF fluorescence was then measured over time using a TECAN M 1000 plate reader (Mannedorf, Switzerland) (excitation at 488 nm; emission at 520 nm). For HyPer measurements, freshly isolated islets were dissociated and transduced for 4 hrs with 200 hyper-encoding adenoviral particles per cell as determined by AdEasy viral titer kit (Stratagene, La Jolla, CA) [25]. Transduced Islet cells were mixed with matrigel and plated in 4 quadrant CellView Dishes (Greinier Bio-One, Monroe, NC) for 72 hrs and imaged using a Zeiss LSM 710 LIVE fluorescence microscope using excitation wavelengths of 488 and 405 while collecting emissions with a long pass 495 nm filter with output to a linear CCD array. Images were acquired using Zeiss ZEN and analyzed with the “ImageJ for Microscopy” Bundle [26]. Islets were maintained at 37°C during the measurements using a Zeiss incubation chamber.

### Redox measurement

Islets were placed under matrigel on a 4 quadrant CellView Dish and imaged using a Zeiss LSM 710 DUO fluorescence microscope using two photon excitation wavelength 720 nm and emission wavelengths of 371–580 nm [27]. Conditions and analysis tools were similar to HyPer experiments above.

### Oxygen Consumption

Oxygen consumption was measured at 37°C from INS-1 cells grown in Seahorse V.7 multiwell culture plates with a companion extracellular O_2_ flux sensor using the Seahorse XF24 analyzer (Seahorse Bioscience, Billerica, MA). Cells were seeded at a density of 50,000 cells/well and cultured for 2 days. Cells were incubated as for insulin secretion and O_2_ consumption measurements were performed in KRB.

### LC-CoA and free CoA

INS-1 cells were grown to approximately 1 million cells/well in 12-well plates and incubated as for insulin release. Cells were exposed to 0.2 mM MOG for up to 30 min and media sampled for insulin release. Cells were then quick frozen in liquid nitrogen and stored at −80°C until processed for LC-CoA analysis. Frozen cells were thawed on ice in 1% trichloroacetic acid containing 3.75 mM DTT. Trichloroacetic acid extracted from the precipitated cells was centrifuged (12,000× g, 3 min), washed 3 times with equal volumes of ether and assayed for total free CoA. The precipitated cells were washed with cold water and hydrolyzed in 3.75 mM K_2_HPO_4_ buffer pH 11.5 at 55°C for 10 min to produce free CoA from membrane associated LC-CoA. Free CoA was then measured enzymatically making use of the α-ketoglutarate dehydrogenase reaction as described previously [28]. Standard curves were produced from varying concentrations of both ether washed free CoA and hydrolyzed LC-CoA in order to account for recovery during sample processing. NADH produced in the assay was then detected by luminescence using NADH-dependent bacterial luciferase as detailed by Peyot et.al. [29].

### Glycerol

Glycerol was measured enzymatically from the same final incubation solutions removed from INS-1 cells to monitor MOG-induced insulin release described above. NADH produced in the assay was then detected by luminescence [29].

### Statistical analysis

Statistical analysis was performed using Student's t test where indicated. Values are plotted as averages +/− SEM.

### Materials

Insulin radioimmunoassay kit was from Millipore (Billerica, MA). Fura and DCFDA were from Invitrogen (Carlsbad, CA). Triacsin C was from ENZO Life Sciences (Farmingdale, NY). MOG, DG Kinase inhibitor and all other chemicals were from Sigma (St. Louis, MO). Enzymes for glycerol and LC-CoA analysis were from Sigma or Roche (Mannheim, Germany). Hyper construct was from Axxora LLC. (San Diego, CA).

## Results

**Figure 1:**
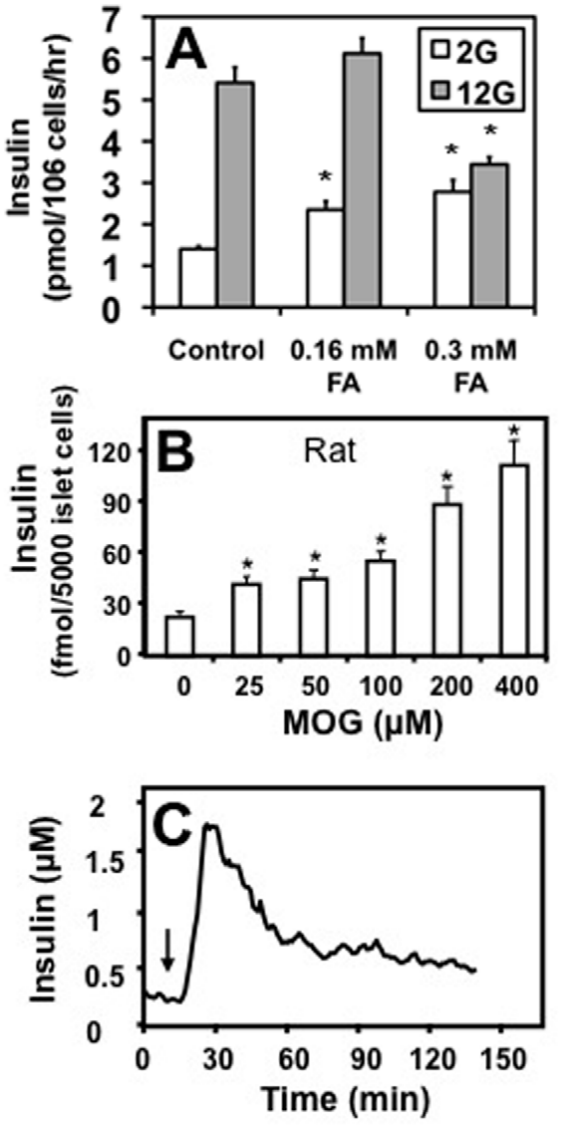
MOG mimicked long-term exposure to glucose and FA by stimulating basal insulin secretion from pancreatic islet cells. A. Long-term exposure to 11 mM glucose and either 0.16 mM or 0.3 mM oleate complexed to FBS (FA) increased basal (2 mM glucose, white bars) and decreased glucose-stimulated insulin release (12 mM glucose, shaded bars) from INS-1 832/13 cells. MOG stimulated basal insulin secretion from dissociated rat islets in a concentration-dependent manner (25–400 µM) (B). C. Perifusion of rat islets demonstrated the pattern of MOG-stimulated basal insulin secretion. Arrow indicates addition of 200 µM MOG to 3 mM glucose. A and B. n = 12 from 3 separate experiments. C. Representative of 3 separate experiments. A and B, *p<0.005 compared to control.

The common technique to create insulin resistance *in vivo* is to feed a high fat-high carbohydrate diet to model animals. The ß-cell malfunction that is induced in this way can also be reproduced *in vitro* by prolonged incubation in media containing high glucose and added FA. Fig. 1A illustrates the consequences of treatment of INS-1 832/13 cells for 18 hours in media containing 11 mM glucose and either 0.16 mM or 0.3 mM oleate complexed to FBS. As has been shown by others [4], [30], this results in a 2-fold increase in secretion at 2 mM glucose (basal) with responsiveness to stimulatory 12 mM glucose diminished by 50 percent. The increase in basal insulin secretion occurred at a lower FA concentration than did the change in glucose-stimulated insulin release. This robust stimulation of basal secretion does not occur acutely but requires several hours of incubation and may involve altered expression of numerous metabolically sensitive proteins [31], [32]. Thus, it is not clear whether this hypersecretion requires altered gene expression or a time-dependent generation of an intracellular product such as LC-CoA, that we have documented previously [33].

To determine whether hypersecretion could be caused acutely, we assessed the effect of several lipid molecules and metabolites on basal secretion. We found a useful tool in MOG that had a concentration dependent ability to increase insulin secretion by isolated rat pancreatic islets (Fig. 1B) at non-stimulatory glucose. Perifusion studies in isolated islets indicated that this effect consisted of a rapid increase followed by a recovery to a new sustained elevated basal level of secretion (Fig. 1C). Similar responses were obtained in INS-1 cells (data not shown).

**Figure 2:**
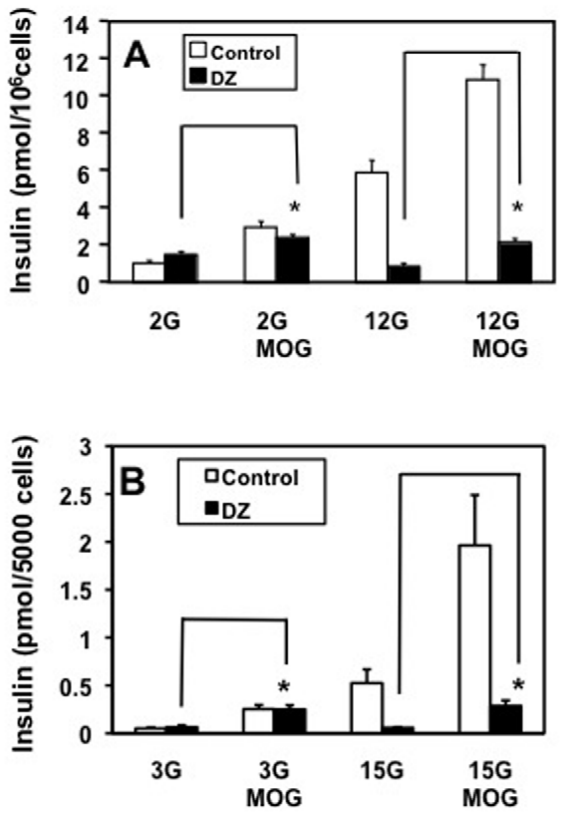
MOG-stimulated basal insulin secretion was independent of the K_ATP_ channel. MOG (0.2 mM) stimulated basal (2 mM glucose) insulin release (white bars) was not prevented by addition of 0.4 mM diazoxide (black bars) in both INS-1 (A) and dissociated rat islet cells (B). MOG enhanced glucose-stimulated insulin release in both INS-1 (12 mM glucose) and islet cells (15 mM glucose) (white bars). Diazoxide blocked glucose-stimulated insulin release with and without MOG (black bars), revealing a sustained increase of basal secretion in the presence of MOG. A. n = 9 in 3 separate experiments. B. n = 6 from duplicate experiments. * p<0.005.

Fuel-induced insulin secretion involves an elevation in cytosolic Ca^2+^, presumably due to closure of the K_ATP_-channel, depolarization and opening of voltage-gated Ca^2+^ channels. Diazoxide, used to prevent closure of the K_ATP_-channels, did not diminish MOG-induced secretion while completely blocking GSIS in INS-1 cells (Fig. 2A) and dissociated rat islets (Fig. 2B). The combination of MOG and stimulatory glucose increased insulin secretion more than either alone. Diazoxide in this case reduced insulin secretion stimulated by glucose in the prescence of MOG to the level of MOG-stimulated basal release (Fig. 2), further indicating that MOG-stimulated insulin secretion was independent of the K_ATP_-channel.

**Figure 3:**
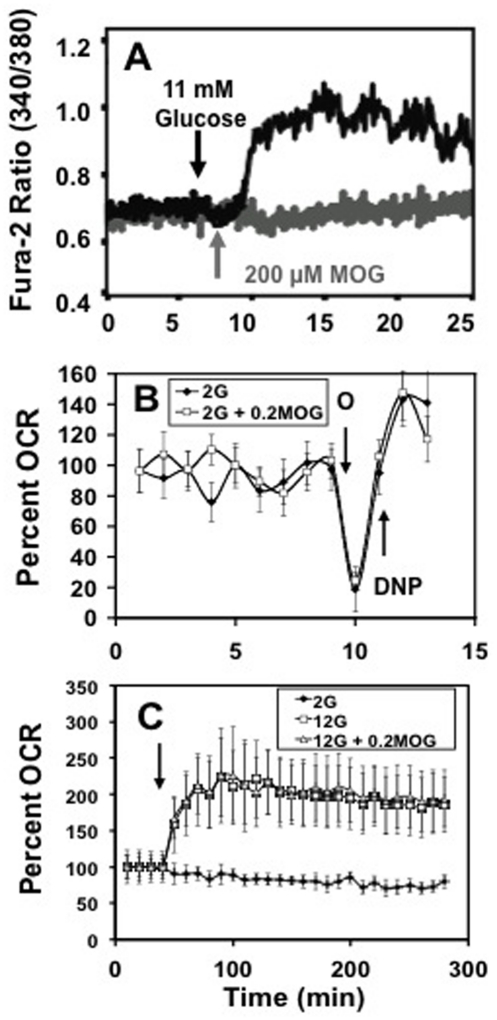
The effect of MOG to stimulate basal insulin secretion was independent of changes in cytosolic Ca^2+^ and oxygen consumption. A. 200 µM MOG did not increase cytosolic Ca^2+^ in dissociated rat islet cells. Arrows indicate addition of 11 mM glucose (black trace) and 200 µM MOG (gray trace). B. 200 µM MOG (white squares) did not affect basal O_2_ consumption in INS-1 cells. Incubation with and without MOG started 30 min prior to O_2_ consumption measurements. Arrows indicate addition of 5 µM oligomycin (O) to inhibit respiration and 100 µM dinitrophenol (DNP) to stimulate maximal respiration. C. 200 µM MOG did not affect glucose-stimulated O_2_ consumption in INS-1 cells. Arrow indicates addition of 2 mM glucose (black diamonds) and 12 mM glucose alone (white squares) or with 200 µM MOG (white triangles). A. Average signal from more than 10 cells per condition. Representative of 3 separate measurements. B and C. n = 4 from single experiment repeated three times.

Consistent with a K_ATP_-channel-independent mode of signaling, there was no change in cytosolic free Ca^2+^ in response to the acute addition of MOG to dissociated ß-cells incubated at basal glucose conditions (Fig. 3A).

Another attribute of GSIS and secretion induced by other stimulatory fuels is an increase in O_2_ consumption. Although we confirmed that stimulatory glucose increased respiration as expected, MOG did not increase O_2_ consumption at 2 mM glucose (Fig. 3B) or increase the stimulated respiration that accompanied GSIS (Fig. 3C). These data indicated that MOG-induced insulin secretion was independent of the consensus signaling pathway involving metabolite-induced stimulation of respiration with increased production of ATP, closure of K_ATP_-channels and entry of Ca^2+^ [34].

**Figure 4:**
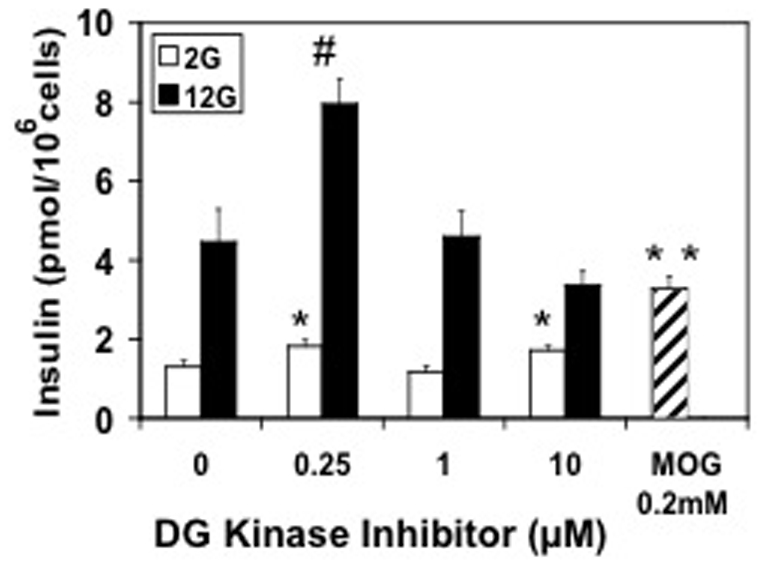
The DG kinase inhibitor R59949, failed to mimic the effects of MOG to stimulate insulin secretion from INS-1 ß-cells at 2 mM glucose. 0.2 mM MOG (hatched bar) increased basal (2 mM glucose) insulin secretion from INS-1 cells 2.5-fold while 12 mM glucose resulted in a 3.5-fold increase (first black bar). The effect of the DG kinase inhibitor R59949 to stimulate basal insulin secretion (white bars) was small and inconsistent over the concentration range tested (0.25–10 µM) compared to the MOG-stimulated increase in basal release (hatched bar). R59949 increased glucose-stimulated insulin release 2-fold at low concentration (0.25 µM) with higher concentrations (1–10 µM) having no stimulatory effect compared to the control (black bars). N = 9 from 3 separate experiments. *p<0.05; **p<0.005 compared to control basal value. #p<0.005 compared to high glucose control.

Protein kinase C involvement in insulin secretion has been documented previously [24]. An earlier study considered MOG an inhibitor of DGK and concluded that its ability to increase insulin secretion was due to this inhibition and the resulting signals generated by accumulated diacylglycerol (DG) [17], [18]. To evaluate this possibility we used a specific inhibitor of DGK, R59949, and found that although it stimulated GSIS at 0.25 µM, it markedly inhibited GSIS at 10 µM, and had little effect on basal secretion under conditions where MOG potently stimulated insulin secretion (Fig. 4). This indicated that simply inhibiting DGK is not sufficient to fully stimulate insulin secretion and suggested that MOG must generate other signals.

**Figure 5:**
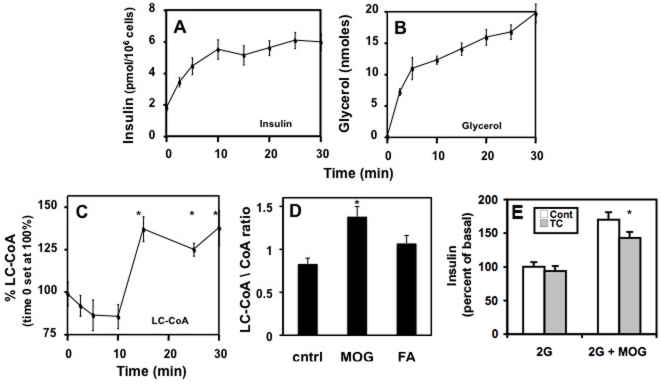
MOG was metabolized by INS-1 ß-cells leading to an increase in glycerol (B), LC-CoA (C) and the LC-CoA/CoA ratio (D). Inhibition of LC-CoA synthetase with triacsin C reduced the stimulatory effect of MOG on basal (2 mM glucose) insulin secretion (E). A. 200 µM MOG increased basal (2 mM glucose) insulin secretion from INS-1 cells in a time-dependent manner. Glycerol release (B) and LC-CoA formation (C) were increased over time from the same cells. D. 200 µM MOG increased LC-CoA: free CoASH ratio to a greater extent than 200 µM oleic acid bound (4∶1 molar ratio) to BSA (FA) compared to the 2 mM glucose control after 1 hr incubation. E. Triacsin C (96 µM) inhibited insulin secretion stimulated by 100 µM MOG by 30% without affecting basal (2 mM glucose) insulin secretion from INS-1 cells. Triacsin C was included in both the preincubation and test conditions. A and B p<0.005 for all. C and E. *p<0.05 n = 9 to 12 from 3–4 different passages of cells. D. *p<0.05 n = 4 from single experiment.

The assessment of MOG as a potential lipid secretagogue was based on abundant evidence that lipids can generate important signals in ß-cells via their metabolism. To determine whether MOG was metabolized we assessed the temporal relationship between insulin secretion (Fig. 5A) and the products of MOG metabolism, glycerol (Fig. 5B), long-chain acyl-CoA (LC-CoA) (Fig. 5C) and the ratio of LC-CoA∶CoASH (Fig. 5D) in INS-1 cells. Oleate that does not stimulate insulin secretion acutely at basal glucose had significantly less effect to elevate LC-CoA and the LC-CoA∶CoASH ratio than MOG (Fig. 5D). Triacsin C, an inhibitor of ACS activity [35], decreased the stimulatory effect of MOG on basal insulin secretion by 30% (Fig. 5E). The possibility that MOG-induced insulin secretion was mediated by LC-CoA (Fig. 5C) or the LC-CoA∶CoASH ratio (Fig. 5D) is consistent with these data. We have previously shown that LC-CoA or a product formed from LC-CoA directly stimulates exocytosis [36]. Glycerol and FFA added in combination to cells does not induce an increase in basal secretion [10], [11], suggesting intracellular metabolism of MOG is required. These data are consistent with the concept that a metabolic product formed from MOG could cause hypersecretion.

**Figure 6:**
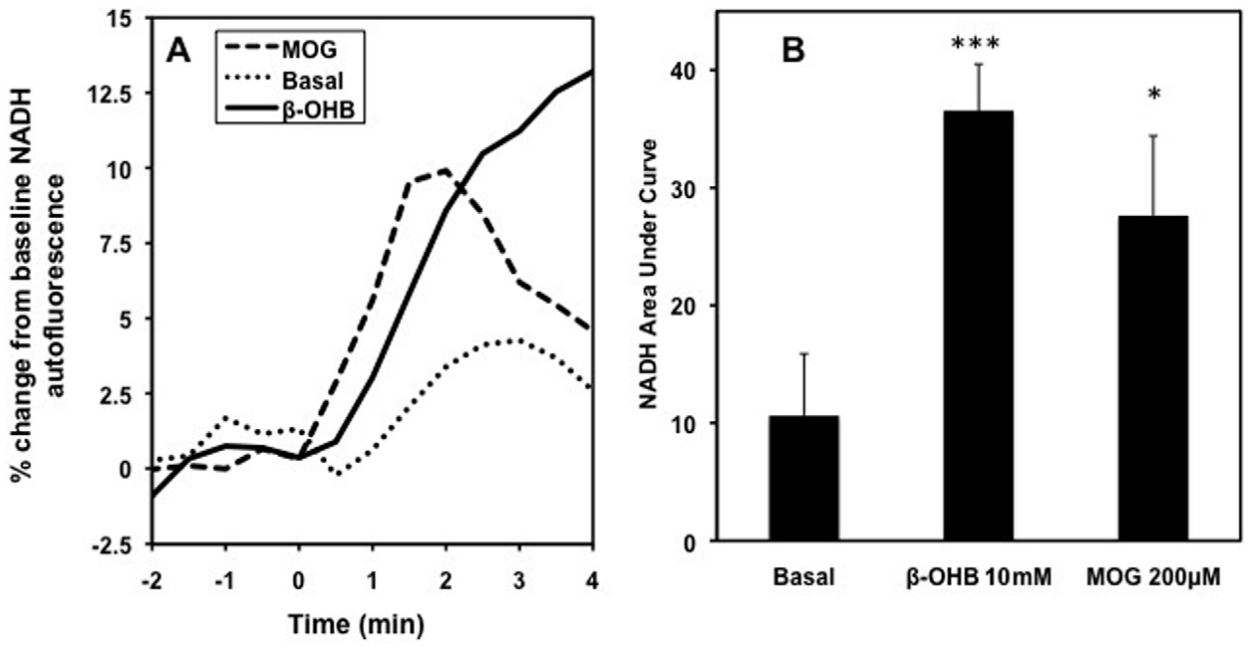
MOG and ß-OHB increased REDOX state in rat pancreatic islets. A. NADH fluorescence was increased in rat islets after addition, at 0 minutes, of 200 µM MOG (dashed line) or 10 mM ß-OHB (solid line) compared to basal 3 mM glucose (circles). Data plotted as a 3 point moving average. B. Area under curve from point of addition to four minutes. Average of 48–55 islets from 3 separate animals. *p<0.05 ***p<0.0001.

The ability of stimulatory glucose and other stimulatory fuels such as ß-OHB to increase redox is well established [21]. ß-OHB is readily transported into the mitochondria where ß-OHB dehydrogenase converts it to acetoacetate using NAD and producing NADH, as we have shown previously [37]. Fig. 6 shows that indeed MOG, like ß-OHB, increased the redox state rapidly and significantly in islets (Fig. 6 A and B). It has been established that ROS generation is dependent on both the mitochondrial redox state and mitochondrial membrane potential (Δ¥) [38].

**Figure 7:**
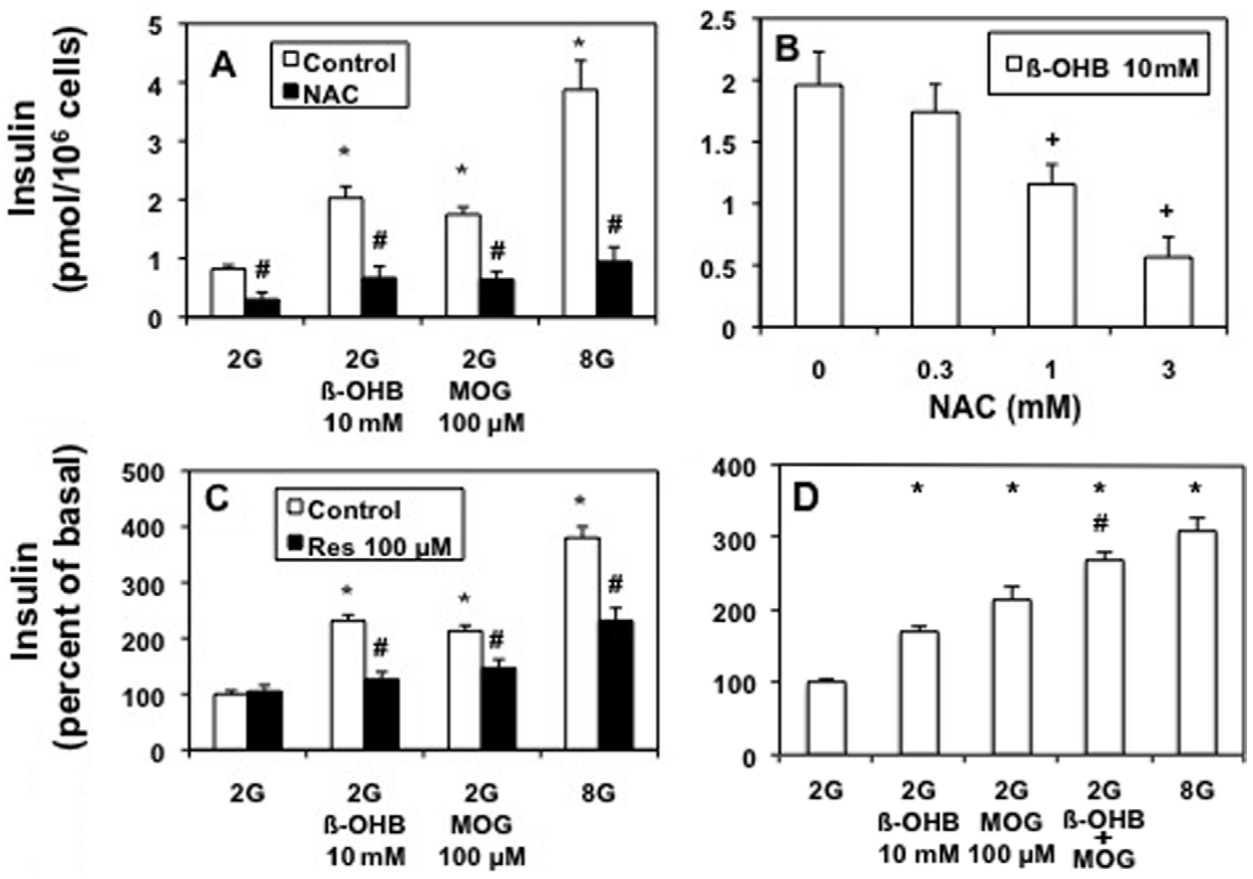
Scavenging ROS with NAC (A, B) or Resveratrol (C) blocked MOG, ß-OHB and glucose-stimulated insulin secretion. The effect of MOG to stimulate basal (2 mM glucose) insulin secretion was not enhanced by the prescence of ß-OHB (D). A. 3 mM NAC (black bars) inhibited 10 mM ß-OHB-, 100 µM MOG- and 8 mM glucose-stimulated insulin secretion from INS-1 cells. B. 10 mM ß-OHB-stimulated insulin secretion was inhibited by NAC in a concentration-dependent manner (0.3–3 mM). C. 100 µM resveratrol (black bars) inhibited 10 mM ß-OHB-, 100 µM MOG- and 8 mM glucose-stimulated insulin secretion from INS-1 cells. D. The increase in insulin secretion stimulated by addition of ß-OHB and MOG together was equal to the sum of their individual effects. A–D. n = 9–12 per condition from 3 separate experiments. * p<0.005 compared to 2G control. A, C. # p<0.005 compared to its own control. B. + p,0.005 compared to no NAC. D. # p<0.005 compared to ß-OHB or MOG added separately.

We next evaluated the possibility that the increased mitochondrial redox state could increase insulin secretion via increased ROS generation. Fig. 7 shows that ß-OHB (at high non-physiological concentrations) stimulated insulin secretion at basal glucose like 100 µM MOG or 8 mM glucose (Fig. 7A). Treatment with the ROS scavenger N-acetyl L-cysteine (NAC) prevented the elevation in basal secretion (Fig. 7A) in a concentration dependent manner (Fig. 7B), implying a ROS-dependent mechanism. Resveratrol, another antioxidant, also inhibited ß-OHB, MOG and glucose induced secretion (Fig. 7C), presumably by scavenging ROS. The increase in insulin secretion stimulated by the combination of ß-OHB and MOG added together was not significantly different to the sum of their individual effects (Fig. 7D). This was also true for the combination of ß-OHB and 8 mM glucose (data not shown). This is in contrast to the enhanced effects of MOG in the presence of stimulatory glucose (Fig. 2).

**Figure 8:**
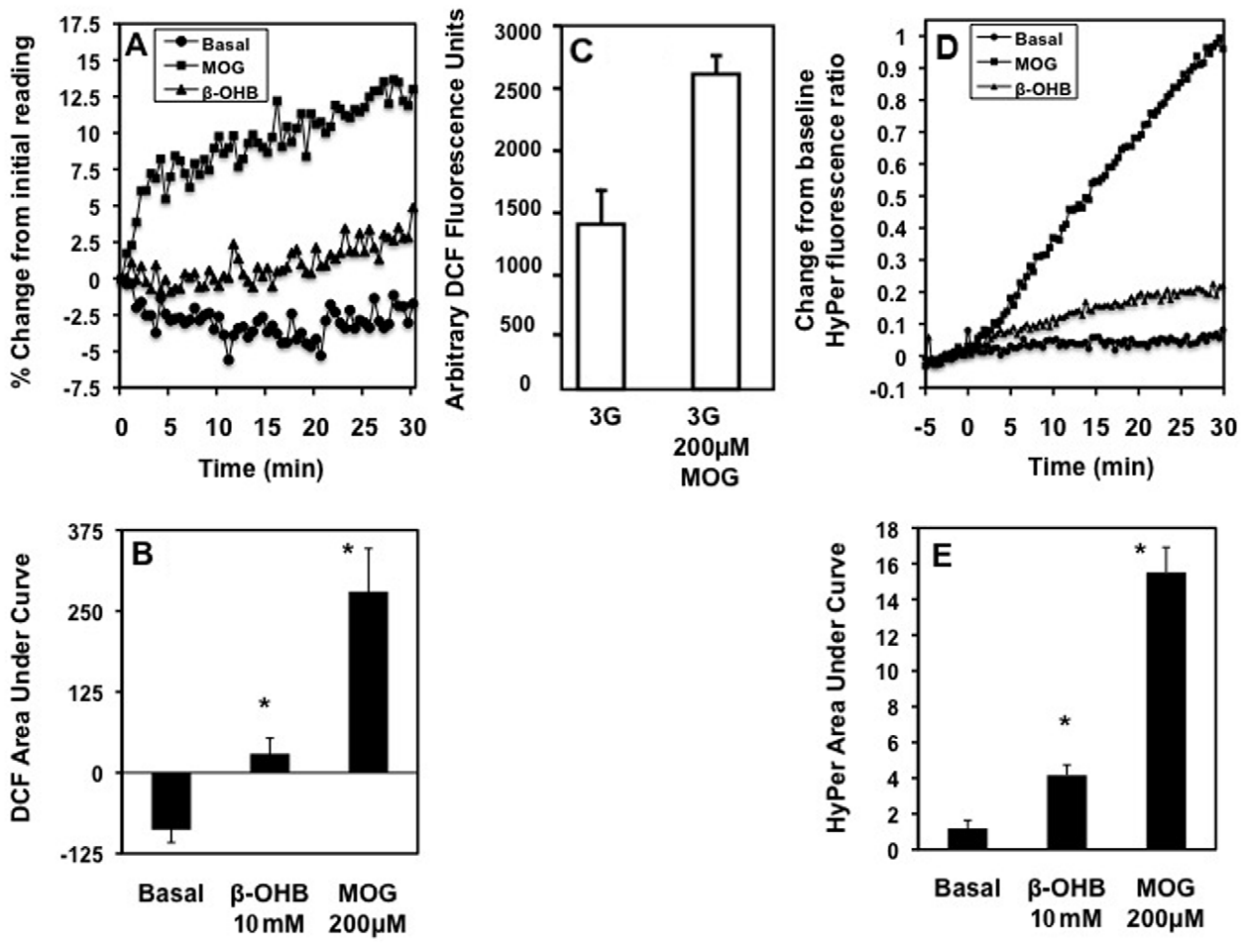
MOG and ß-OHB generated ROS in insulin secreting cells. A. Both 200 µM MOG (squares) and 10 mM ß-OHB (triangles) increased ROS in INS-1 cells compared to basal (2 mM glucose) alone (circles). B. Area under curve of panel A. 200 µM MOG increased ROS generation in dissociated rat islets (C) compared to 3 mM glucose controls as measured with DCF fluorescence. D. 200 µM MOG (squares) and 10 mM ß-OHB (triangles) increased ROS generation in dissociated islet cells expressing the cytosolic HyPer protein compared to the basal (2 mM glucose) control as measured by changes in fluorescence ratio. E. Area under curve of D. A, B. n = 16 from 4 separate passages of cells. C. Results are typical of three separate experiments. D, E. Average of 14–33 cells from 3 separate animals. (A–C). ROS monitored for 1 hr in cells loaded with CM-H_2_DCFA using a Tecan fluorescence platereader. (D–E). HyPer fluorescence ratio in dissociated islet cells monitored using a Zeiss 710 LIVE fluorescence microscope at 20× magnification. B and E, * p<0.005% compared to basal.

We previously demonstrated that provision of either exogenous H_2_O_2_ or diethyl maleate, which raises intracellular H_2_O_2_ levels, stimulated insulin secretion [39]. In addition, exogenous antioxidants such as cell permeable catalase and NAC inhibited GSIS and intracellular H_2_O_2_ accumulation [39]. More recently inhibition of isoprenylcysteine carboxyl methyltransferase activity in INS-1 832/13 cells was shown to inhibit both glucose-induced ROS production and insulin secretion [40]. In Fig. 8 we show that MOG and ß-OHB, like glucose, generated ROS in INS-1 cells (Fig. 8A and B). We also document that MOG- and ß-OHB-induced ROS in dissociated rat islets (Fig. 8C, D and E). The increase in ROS occurred rapidly with MOG compared to ß-OHB as measured by increased DCF fluorescence (8A and B) and increased Hyper fluorescence ratio (Fig. 8D and E).

## Discussion

Using MOG and the mitochondrial reductant ß-OHB, we have identified ROS as a novel obligatory messenger for insulin secretion, independent of the consensus pathway of GSIS that involves increased respiration, K_ATP_ channel closure, and elevated cytosolic Ca^2+^. MOG rapidly and robustly mimicked the effects of long-term exposure to elevated FA and glucose and the condition described as glucolipoxity, manifested as basal insulin hypersecretion.

The possible novel signals for insulin secretion derived from MOG metabolism include lipid products such as LC-CoA (34) and DG [17], [18], ROS [39], action through membrane receptors such as GPR40 [41] or protein acylation effects of the elevated LC-CoA. We can rule out non-specific cell damage because islets treated with MOG recovered fully following overnight incubation in normal media (data not shown) and exhibited normal respiratory responses to glucose (Fig. 3C). The fact that MOG metabolism gave rise to increases in glycerol and LC-CoA exhibiting different time-courses was unexpected (Fig. 5 B and C). It may be that LC-CoA is rapidly esterified to other lipid moieties inside the cell while the lack of glycerol kinase in the ß-cell [42] precludes this re-esterification to glycerol itself. Thus it is predicted that MOG gives rise to a host of other lipid moieties in the ß-cell whose identification is beyond the scope of this study. A role for LC-CoA, either directly or indirectly through lipid esterification or protein acylation, is indicated as a result of the partial inhibition of MOG-induced basal insulin secretion using triacsin C, an acyl CoA synthetase inhibitor. Since it was possible to block the stimulatory effects of MOG, ß-OHB and glucose with the ROS scavengers NAC and resveratrol, we concluded that ROS was the obligatory signal, whereas the other putative mediators may be secondary. It is interesting that 200 µM MOG stimulates a greater production of ROS in pancreatic ß-cells than 10 mM ß-OHB. The metabolism of MOG results in a global increase in LC-CoA in the cell leading presumably to other lipid moieties while ß-OHB is metabolized solely in the mitochondria of these cells. The contributions of cytosolic versus mitochondrial sources of ROS measured as a result of MOG and other nutrient metabolism is under investigation.

It is interesting to speculate why FA addition requires much more time to cause basal hypersecretion than MOG. Several possibilities may be considered. First, there is control of FA access to the cell by albumin and acyl CoA synthases. High physiological concentrations of albumin prevent rapid uptake of large quantities of FA in excess of the ability of acyl CoA synthases to activate them. This idea is supported by our previous studies showing that only a small increase in LC-CoA occurs in response to FA acutely but LC-CoA increases following overnight incubation [33], [43]. Second, there are two possible pathways for MOG metabolism: via MGL that generates FA and glycerol and via monoacylglycerol acyl transferase (MGAT) to generate DG. The relative roles of MGL and MGAT have not been assessed in the ß-cell. However, both may occur and the implications of each may differ: DG acting through a PKC signaling cascade and LC-CoA directly stimulating exocytosis [36] as well as acting on a variety of steps involved with energy metabolism.

We have previously demonstrated that LC-CoA stimulates exocytosis at low Ca^2+^ concentrations [36] and activates the K_ATP_ channel [33], [44], [45], [46], [47] leading to impaired Ca^2+^ signaling [48]. Interesting recent studies have documented reduced ß-cell excitability following elevation of endogenous saturated LC-CoA in intact pancreatic ß-cells [49] and modulation of ß-cell Na^+^-Ca^2+^ exchangers by LC-CoA [49], [50]. In addition, LC-CoA has been shown to overcome inhibition of carnitine palmitoyl transferase-1 by malonyl CoA [51], and inhibit the adenine nucleotide translocase [52], [53], both of which are important in stimulation of glucose-induced insulin secretion. Thus, increased LC-CoA after MOG exposure is a potential candidate for mediating some of the observed aberrant effects on secretion. The effect of triacsin C to partially block the stimulation of basal insulin secretion by MOG provides supporting evidence for a role for LC-CoA in the development of lipid-induced basal hypersecretion.

It is not clear whether these effects of MOG are of physiological importance. MOG caused a twofold increase in basal insulin secretion at concentrations as low as 25 µM (Fig. 1). Monoglycerides are normal products of TG degradation by tissue-associated lipoprotein lipase [54] and in the intestine where a remnant molecule of 2-monoglyceride is transported into the enterocytes with two FA liberated from TG. In the past 30 years, monoglycerides have been increasingly added to foods as emulsifiers or preservatives. However, relevance to human physiology and the obesity and diabetes epidemic cannot be implied, as circulating concentrations were not found and may be considerably lower than concentrations tested. It is possible, however, that long term exposure to low concentrations of MOG may elevate basal insulin secretion like long term exposure to GL.

The key findings from these data are that basal hypersecretion can be induced by exposure to MOG in the absence of elevated glucose without increasing cytosolic Ca^2+^ or O_2_ consumption in human islets, rodent islets and INS-1 cells. It should be noted that an increase in redox without an increase in respiration implies excess mitochondrial substrate supply (NADH) that leads to increased ROS production. During the same interval we have shown that MOG was metabolized, increased the redox state and generated ROS. Scavenging ROS prevented all of the acute MOG effects on secretion indicating that ROS was an obligatory signal for insulin secretion. Mimicking the MOG-induced redox changes with ß-OHB or inducing ROS changes by other means [39] also increased insulin secretion. Thus, ROS may act synergistically with other putative signals such as LC-CoA, Ca^2+^ and DG but basal hypersecretion does not appear to occur without the ROS signal.

ROS may also play a role in elevated basal insulin secretion resulting from more traditional means of inducing GL. In this regard gene expression profiling of clonal pancreatic ß-cells cultured (24–72 hrs) under conditions of elevated glucose and FA has been performed in the mouse cell line MIN6 using oleate [55] and in INS-1 832/13 using palmitate [56]. Both studies revealed a host of changes in metabolic enzymes affecting lipid handling. Both studies demonstrated an increase in ROS production and impaired insulin secretion in that basal secretion was elevated and glucose-stimulated insulin release was inhibited. Investigators using the MIN6 model also used NAC (1 mM) to scavenge ROS. In this case they were able to reduce by 70% the number of oleate-induced downregulated genes. NAC was reported not to alter insulin secretion from the oleate treated cells, however insulin content of cells treated with NAC was increased 2-fold compared to oleate alone. NAC therefore could be said to have increased insulin exocytosis when analyzed as insulin release as a percent of content.

The rapidity of MOG effects may provide a novel model to examine the role of elevated lipids on insulin secretion without concern for the alterations in gene expression that may occur over the hours required to achieve similar elevations in secretion by GL.

Furthermore, the acute elevation of intracellular lipids by MOG implied by the large increase in LC-CoA, independent of stimulatory glucose, may provide a useful model to study the role of lipid signaling during insulin resistance and may lead to identification of important lipid and protein signals that modulate secretion in the ß-cell.
